# Efficient anaerobic consumption of D-xylose by *E. coli* BL21(DE3) via *xylR* adaptive mutation

**DOI:** 10.1186/s12866-021-02395-9

**Published:** 2021-12-06

**Authors:** Jung Min Heo, Hyun Ju Kim, Sang Jun Lee

**Affiliations:** Department of Systems Biotechnology, Chung-Ang University, Anseong, 17546 Republic of Korea

**Keywords:** BL21(DE3), Anaerobic fermentation, D-xylose, XylR, Adaptive mutation

## Abstract

**Background:**

Microorganisms can prioritize the uptake of different sugars depending on their metabolic needs and preferences. When both D-glucose and D-xylose are present in growth media, *E. coli* cells typically consume D-glucose first and then D-xylose. Similarly, when *E. coli* BL21(DE3) is provided with both D-glucose and D-xylose under anaerobic conditions, glucose is consumed first, whereas D-xylose is consumed very slowly.

**Results:**

When BL21(DE3) was adaptively evolved via subculture, the consumption rate of D-xylose increased gradually. Strains JH001 and JH019, whose D-xylose consumption rate was faster, were isolated after subculture. Genome analysis of the JH001 and JH019 strains revealed that C91A (Q31K) and C740T (A247V) missense mutations in the *xylR* gene (which encodes the XylR transcriptional activator), respectively, controlled the expression of the *xyl* operon. RT-qPCR analyses demonstrated that the XylR mutation caused a 10.9-fold and 3.5-fold increase in the expression of the *xylA* (xylose isomerase) and *xylF* (xylose transporter) genes, respectively, in the adaptively evolved JH001 and JH019 strains. A C91A adaptive mutation was introduced into a new BL21(DE3) background via single-base genome editing, resulting in immediate and efficient D-xylose consumption.

**Conclusions:**

Anaerobically-adapted BL21(DE3) cells were obtained through short-term adaptive evolution and *xylR* mutations responsible for faster D-xylose consumption were identified, which may aid in the improvement of microbial fermentation technology.

**Supplementary Information:**

The online version contains supplementary material available at 10.1186/s12866-021-02395-9.

## Background

Recent microbiome studies have increasingly focused on the correlation between added sugars in foods consumed by humans and changes in microbial communities [1]. Intestinal microbes can uptake and metabolize sugars and supply beneficial metabolites such as short-chain fatty acids as energy sources to intestinal epithelial cells [2–4]. D-xylose is abundant in fiber and is rarely absorbed by the gastrointestinal tract. Therefore, this compound is often used as a sweetener in food and beverages instead of sugars (e.g., cane sugar, high fructose corn syrup), which can cause metabolic diseases such as diabetes and obesity [5]. Nonetheless, these sweeteners can affect the structure of the oral and intestinal microbial community [6–8]. Therefore, identifying the mechanisms by which microorganisms absorb and metabolize D-xylose under anaerobic conditions has garnered increasing attention in recent years.

*E. coli* has a native xylose transport system that enables these bacteria to metabolize xylose [9, 10]. D-xylose is known to be transported into *E. coli* cells through the D-xylose/proton symporter XylE and the XylFGH ATP-dependent ABC transporter [11, 12]. The absorbed D-xylose is converted to D-xylulose by xylose isomerase XylA, phosphorylated to D-xylulose-5-phosphate by xylulosekinase XylB, and linked to glycolysis through the pentose phosphate pathway [13].

The *xylFGH* and *xylAB* genes, which respectively encode xylose uptake and metabolism-related enzymes, are co-regulated by the XylR transcriptional factor, as well as the intracellular cyclic AMP (cAMP) concentration. XylR is a transcriptional activator that directly regulates the xylose operon by binding to the promoter of the regulatory region in the presence of D-xylose (i.e., the inducer) [14]. However, D-xylose metabolism is inhibited by glucose in many microorganisms including *E. coli*, a phenomenon known as carbon catabolite repression (CCR) [15, 16]. In the presence of D-glucose, CCR inhibits the uptake of other sugars such as D-xylose or lactose. This results in a phenomenon referred to as diauxic growth, whereby other sugars are consumed once D-glucose is fully depleted [17–20]. In the absence of D-glucose (i.e., the preferred carbon source), sugars such as lactose, L-arabinose, and D-xylose are consumed sequentially, depending on the sugar preference [21]. Inhibition of the consumption of other phosphotransferase system (PTS)-sugars by glucose can also be interpreted as an inducer exclusion mechanism [22]. In the presence of glucose, the phosphate group of glucose-specific enzyme IIA [EIIA (glc)] is transferred to the incoming sugar, and EIIA exists in an unphosphorylated form and binds to non-PTS sugar permeases. Therefore, the transport of non-PTS sugars is inhibited [16, 23]. Co-utilization studies of xylose and glucose in *E. coli* have also been performed by co-culturing a xylose transporter-deficient strain and a strain in which glucose transport-related genes (e.g., *ptsG, glk,* and *manZ*) were deleted [24, 25]. Additionally, other studies have reported the use of a cyclic AMP-independent CRP mutant to avoid catabolic repression [26, 27].

The *xylFGH* gene encodes the ABC transporter involved in xylose uptake. However, when this gene is deleted via adaptive evolution, xylose is absorbed through GatC, an alternative transporter [28]. In another study, the uptake and metabolism of xylose was enhanced using cell culture techniques coupled with evolutionary engineering, and a *xylR* mutation was identified in a mutant strain that readily consumed D-xylose [29].

Our study confirmed that the rate of xylose consumption after glucose depletion varied among *E. coli* wild-type strains when both glucose and xylose were present in the growth medium. *E. coli* strain BL21(DE) consumed D-xylose very slowly compared to other strains. Therefore, experimental evolution was performed to increase the D-xylose consumption rate of this strain. Moreover, we demonstrated the potential use of strain BL21(DE3) as a model for the development of microorganisms that can quickly consume D-xylose, which is an important milestone for the advancement of fermentation technology.

## Results

### Different D-xylose consumption rates in *E. coli* strains under anaerobic conditions

**Fig. 1:**
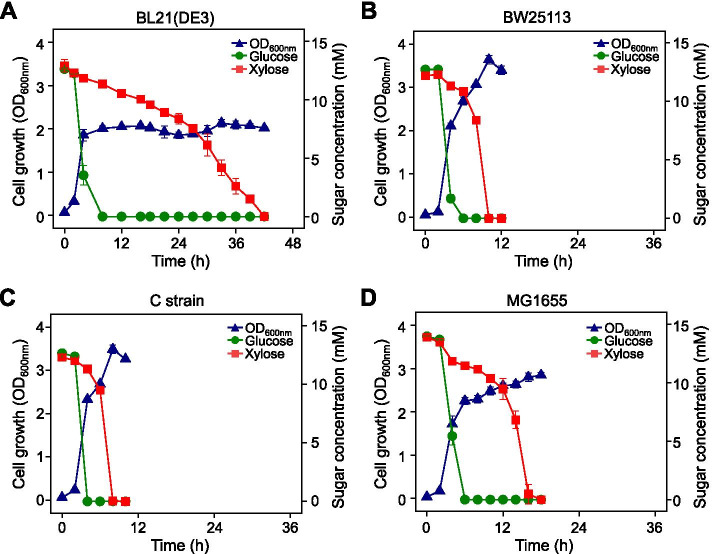
Cell growth and sugar consumption profiles of wild-type *E. coli* strains in anaerobic conditions. Both D-glucose (12.5 mM) and D-xylose (12.5 mM) were added to the fermentation medium as carbon sources. **A** BL21(DE3); **B** BW25113; **C** C strain; **D** MG1655

*E. coli* BL21(DE3), BW25113, C strain, and MG1655 strains were anaerobically grown in a fermentation medium supplemented with D-glucose (12.5 mM) and D-xylose (12.5 mM). All four strains consumed D-glucose and appeared to fully consume the D-glucose within 4–6 h (Fig. 1). Cells began to uptake D-xylose after depleting the D-glucose; however, the D-xylose consumption rate varied in a strain-dependent manner. BW25113 and C strain fully depleted the D-xylose 4 h after consumption of D-glucose. After depleting the D-glucose, it took 10 and 36 h for the MG1655 and BL21(DE3) strains to consume the D-xylose, respectively. The BL21(DE3) strain exhibited the longest D-xylose consumption delay after D-glucose depletion. Moreover, even when the cells were cultured in D-xylose-only media, the D-xylose consumption rate of the BL21(DE3) strain was slower than that of the other strains (Fig. S1).

### Accelerated anaerobic growth of BL21(DE3) strains through adaptive evolution

**Fig. 2:**
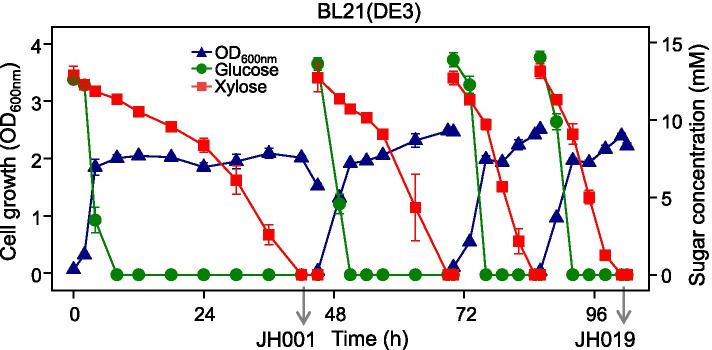
Serial transfer of *E. coli* BL21(DE3) in anaerobic fermentation media. The JH001 and JH019 strains were obtained at the first and fourth passages, respectively, once D-xylose was fully depleted

Our study sought to obtain BL21(DE3) cells with an increased D-xylose consumption rate through adaptive evolution by serially transferring cultures to fresh fermentation media. BL21(DE3) cells were anaerobically grown in a fermentation medium containing D-glucose (12.5 mM) and D-xylose (12.5 mM). The growth rate and D-glucose consumption rate of the bacteria did not change significantly after several transfers; however, the maximum D-xylose consumption rate increased gradually. The D-xylose consumption rate was 0.6 mM/h in the first passage, but gradually increased to 0.8, 1.3, and 1.4 mM/h in each subsequent passage (Fig. 2). Cultures of the 1st and 4th passages were spread on LB agar plates to isolate pure colonies. The progeny strains JH001 and JH019 were obtained after anaerobically culturing the newly obtained colonies in the same medium. Notably, these progeny strains consumed D-xylose faster after full D- glucose depletion compared to the parent strain.

**Fig. 3:**
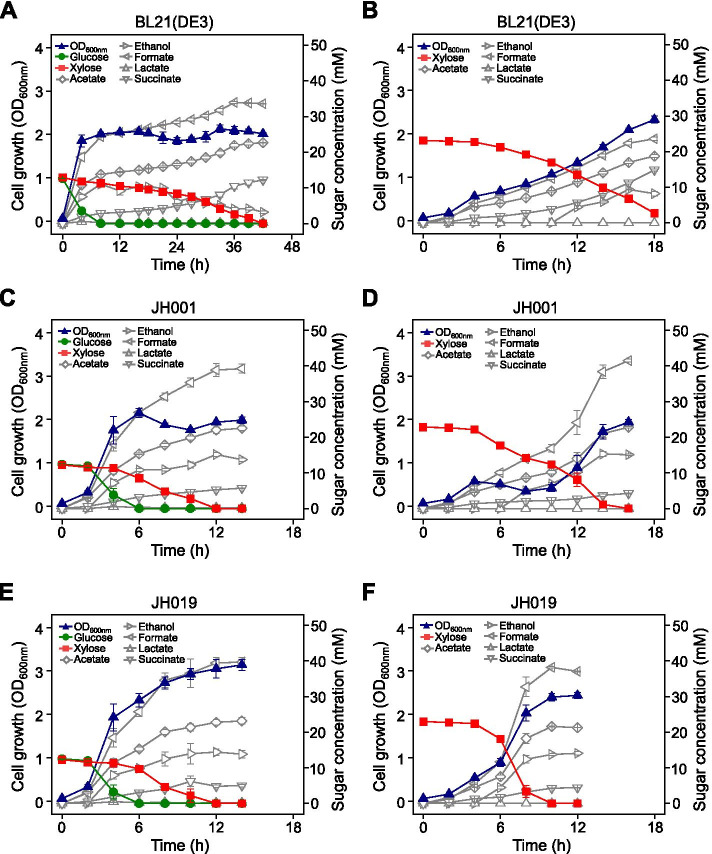
Anaerobic cell growth and fermentation profiles of the BL21(DE3) *E. coli* strain and adaptively evolved strains in fermentation medium containing both D-glucose (12.5 mM) and D-xylose (12.5 mM), or fermentation medium containing D-xylose only (25 mM). **A** BL21(DE3) in D-glucose + D-xylose, **B** BL21(DE3) in D-xylose, **C** JH001 in D-glucose + D-xylose, **D** JH001 in D-xylose, **E** JH019 in D-glucose + D-xylose, and **F** JH019 in D-xylose

The maximum D-xylose consumption rate of the wild-type BL21(DE3) strain was 1.1 mM/h in D-glucose- and D-xylose-supplemented anaerobic media. In contrast, the D-xylose consumption rates of the JH001 and JH019 rates were 1.9 mM/h and 2.9 mM/h, which represented 1.7- and 2.7-fold increases compared to the BL21(DE3) strain (Table 1). Moreover, the JH001 strain exhibited an increased D-xylose consumption rate but its cell growth was not significantly increased (Fig. 3C). However, strain JH019 showed increased cell growth (Fig. 3E).

**Table 1:** Specific growth rate and xylose consumption rate, and fermentation profiles of wild-type BL21(DE3) strain and adapted strains with D-glucose + D-xylose, and D-xylose

Added sugar (mM)	Strain	Fermentation time (h)^a^	Specific growth rate (μ) (fold)	Maximum xylose consumption rate (mM/h) (fold)	Metabolites (mM)				
					Acetate	Ethanol	Formate	Lactate	Succinate
Glucose (12.5) +Xylose (12.5)	BL21(DE3)	42	0.88 ± 0.02 (1.0)	1.1 ± 0.0 (1.0)	22.7 ± 0.1	3.2 ± 0.4	33.6 ± 0.1	0.2 ± 0.0	12.2 ± 0.2
	JH001	12	0.85 ± 0.03 (1.0)	1.9 ± 0.2 (1.7)	22.0 ± 0.9	15.2 ± 0.6	38.9 ± 1.8	0.2 ± 0.0	5.3 ± 0.1
	JH019	12	0.91 ± 0.07 (1.0)	2.9 ± 0.6 (2.7)	22.8 ± 0.8	14.4 ± 0.9	39.4 ± 1.5	0.2 ± 0.0	4.8 ± 0.3
Xylose (25)	BL21(DE3)	21	0.59 ± 0.04 (1.0)	2.0 ± 0.0 (1.0)	19.4 ± 0.3	7.4 ± 0.1	23.4 ± 0.4	ND^a^	17.9 ± 0.1
	JH001	16	0.63 ± 0.03 (1.1)	3.7 ± 0.6 (1.9)	22.8 ± 0.2	15.1 ± 0.2	41.5 ± 0.4	ND	4.3 ± 0.1
	JH019	10	0.60 ± 0.02 (1.0)	7.4 ± 0.6 (3.7)	21.6 ± 0.1	13.7 ± 0.3	38.2 ± 0.2	ND	4.3 ± 0.1

^a^Fermentation time (h) when glucose plus xylose or xylose were completely consumed

^a^*ND* Not detected

In the medium containing D-xylose only, the adaptively evolved strains JH001 and JH019 exhibited a faster D-xylose consumption compared to BL21(DE3) (Fig. 3). Concretely, the BL21(DE3) strain had a maximum D-xylose consumption rate of 1.98 mM/h, whereas the JH001 strain exhibited an increased rate of 3.69 mM/h. Moreover, when the JH001 strain was cultured in D-xylose-supplemented medium, the D-xylose was consumed between 4 and 10 h, but cell growth was considerably slower (Fig. 3D). In contrast, the maximum D-xylose consumption rate of the JH019 strain increased to 7.36 mM/h and there were no cell growth delays.

### Variation in fermentation products in adapted BL21(DE3) cells

The difference between the wild-type and adaptively evolved strains was confirmed based on their organic acid and ethanol output during fermentation. When provided with both D-glucose and D-xylose, there was no significant difference in the amount of acetate, formate, and lactate produced by the bacterial strains. However, while the wild-type strain produced 3.2 mM of ethanol, the adaptively evolved strains produced 15.2 and 14.4 mM. Conversely, the JH001 and JH019 strains produced 5.3 and 4.8 mM of succinate, respectively, whereas the wild-type strain produced 12.2 mM.

When provided with D-xylose only, neither the wild-type nor the adaptively evolved strains produced lactate, and acetate production was not significantly different. Moreover, similar to the D-glucose + D-xylose condition, ethanol production was further increased and succinate decreased in the adaptively evolved JH001 and JH019 strains (Table 1).

### Identification of adaptive mutations in the evolved strains via genome sequencing

Whole-genome sequencing analysis of the adaptively evolved strains with increased D-xylose consumption identified a C91A point mutation (Q31K missense in XylR protein) in the *xylR* gene of JH001 strain, as well as a C740T substitution (A247V) in the *xylR* gene and IS (insertion sequence) insertion in the open reading frame of the *carB* gene of the JH019 strain (Table 2). Given that D-xylose cannot be consumed in a *xylR* null mutation background (Fig. S3), we assumed that the *xylR* point mutations represented a gain of function mutation responsible for faster D-xylose uptake in the adaptively evolved strains. Since *xylR* encodes a transcriptional activator, the expression of the xylose operon was also investigated (see below).

**Table 2:** Genomic analysis of *E. coli* BL21(DE3) adapted strains

Strain	Genotype	Reads	Bases	Reads (trimmed)	Bases (trimmed)	Avg. length (trimmed)	Reads matched	% Reads matched	Fraction of reference covered	Avg. coverage
JH001	*xylR* C91A (Q31K)	24,611,910	6,177,589,410	21,909,198	3,353,116,156	153	19,789,604	90	1	664.35
JH019	*xylR* C740T (A247V), *carB*::IS1	17,170,757	3,823,379,807	14,945,996	2,196,575,011	147	14,674,322	98	1	473.06

### Transcript analysis of the xylose operon in adaptively evolved BL21(DE3) strains

**Fig. 4:**
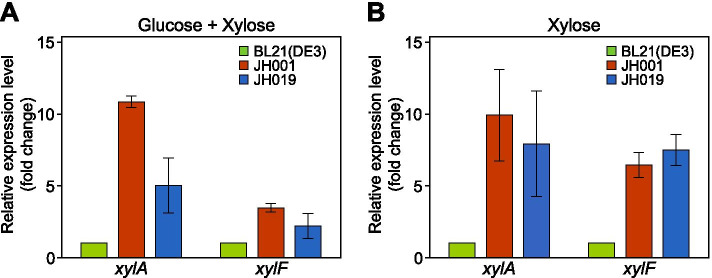
Transcript analysis of *xylA* and *xylF* genes in *E. coli* BL2(DE3) adapted strains using qRT-PCR. **A** Cells grown in fermentation media containing both D-glucose (12.5 mM) and D-xylose (12.5 mM), **B** Cells grown in fermentation media containing D-xylose only (25 mM)

qRT-PCR was conducted to confirm whether the expression of the xylose operon was enhanced in adapted cells carrying *xylR* point mutations. The expression levels of the *xylA* and *xylF* genes (which encode xylose isomerase and xylose ABC transporter, respectively) were compared between the wild-type and adaptively-evolved BL21(DE3) strains grown in a fermentation medium containing both D-glucose (12.5 mM) and D-xylose (12.5 mM). Compared to BL21(DE3), the expression of the *xylA* and *xylF* genes in the JH001 strain were upregulated 11- and 3-fold, respectively. Similarly, in the case of the JH019 strain, the expression levels of the *xylA* and *xylF* genes increased 5- and 2-fold compared to the wild-type strain, respectively (Fig. 4A). When each strain was grown in fermentation media containing only D-xylose (25 mM), the JH001 and JH019 cells exhibited significantly elevated transcript levels of the *xylA* and *xylF* genes, which were at least 5 times higher than those in the wild type BL21(DE3) strain (Fig. 4B). These results suggest that D-xylose transporting and metabolizing enzymes are highly expressed in adapted BL21(DE3) cells carrying *xylR* adaptive mutations.

### Increased D-xylose consumption rate by *xylR* single point genome editing

**Fig. 5:**
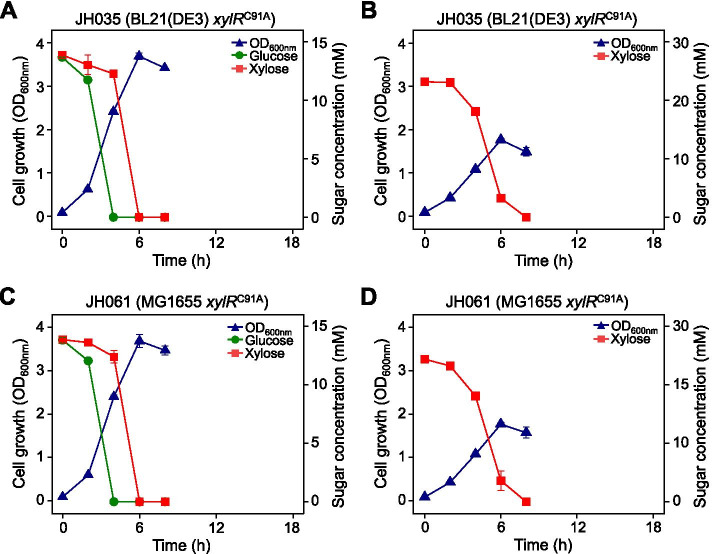
Anaerobic cell growth and sugar consumption profiles of *E. coli* cells carrying the newly introduced *xylR* C91A mutation. **A** JH035 (= BL21(DE3) *xylR* C91A) cells grown in D-glucose + D-xylose medium, **B** JH035 in D-xylose only medium, **C** JH061 (= MG1655 *xylR* C91A) in D-glucose + D-xylose medium, **D** JH061 in D-xylose only medium

The single point mutation (C91A) of the *xylR* gene identified in the adaptively evolved JH001 strain was introduced into the genome of the BL21(DE3) and MG1655 wild-type strains via the target-mismatched CRISPR/Cas9 method [30]. When BL21(DE3) XylR^Q31K^ cells were grown in fermentation media containing both D-glucose and D-xylose, D-xylose was completely consumed 2 h after D-glucose depletion (Fig. 5A). In xylose-only fermentation medium, it took 21 h for the BL21(DE3) strain to fully deplete the D-xylose, whereas BL21(DE3) XylR^Q31K^ cells completely consumed the D-xylose in 6 h (Fig. S1A, Fig. 5B). Similar results were also observed in MG1655 wild-type and MG1655 XylR^Q31K^ cells (Fig. 5C and D). These results indicate that the XylR^Q31K^ mutation is responsible for the faster growth of the evolved BL21(DE3) cells in the fermentation medium through enhanced D-xylose transport and metabolism.

Additionally, to confirm whether the *carB* gene inactivation also identified in the JH019 strain affects the D-xylose consumption rate, a *carB* deletion mutation was introduced into the BL21(DE3) wild-type strain and the adaptively evolved strain JH001. D-xylose consumption rates were accelerated when the *carB* gene deletion was introduced into the BL21(DE3) cells (Fig. S4A and S4B). The *carB* deletion mutation improved the D-xylose consumption rate of the JH001 strain when the cells were grown in a fermentation medium containing only D-xylose (Fig. S4D). These results indicated that the *carB* mutation enhanced anaerobic cell growth in the D-xylose medium.

## Discussion

Although *E. coli* has been widely isolated from gut samples (i.e., anaerobic conditions), the physiology and metabolism of this model microorganism have been extensively studied under aerobic conditions, as these conditions are more easily implemented in laboratories. The priority at which different sugars such as D-glucose, lactose, L-arabinose, and D-xylose are consumed follows a hierarchical order, and carbon source preference is known to be regulated by intracellular cAMP-CRP and transcription factors [16, 31, 32].

The bacterial strains studied herein exhibited diauxic growth, whereby D-xylose is consumed once D-glucose is fully depleted under anaerobic conditions. However, the time required for D-xylose consumption after rapid glucose consumption varied among different *E. coli* strains. Particularly, the BL21(DE3) strain exhibited the longest xylose consumption delay (Fig. 1).

Upon analyzing the genomes of strains JH001 and JH019 obtained by modifying the BL21 (DE3) strain, gain-of-function mutations (Q31K and A247V) in the *xylR* gene were identified. Sievert et al. (2017) recently reported that the expression of the D-xylose operon and subsequent D-xylose consumption were enhanced by R121C and P363S mutations in the *xylR* gene, which might induce compact folding of XylR and increase the DNA binding affinity of XylR, respectively [29]. In our study, we introduced a R121C mutation identified by Sievert et al. (2017) in the *xylR* gene of BL21(DE3) cells, and observed an accelerated consumption of D-xylose after D-glucose consumption. However, the D-xylose consumption rate of XylR^R121C^ cells was not faster than that of XylR^Q31K^ cells. These results indicate that the most suitable adaptive mutations can vary depending on the strain background (Fig. S5).

The Q31K and A247V mutations obtained in our study belong to subdomain 1 of XylR protein [33], and it is assumed that these mutations might be involved in XylR dimerization (Fig. S2). The transcriptional expression level of *xylA* and *xylF* genes was higher in the *xyl* operon of the JH001 and JH019 strains, indicating that the *xylR* mutations were responsible for the accelerated uptake and metabolism of D-xylose (Fig. 4).

Although no mutations have been reported in the xylose operon to date [34, 35], we revisited the nucleotide sequences of the xylose operon in the genome (GenBank accession No. CP001509.3) of BL21(DE3) and identified a single base deletion in the open reading frame of the *xylG* gene*,* which can result in C-terminal truncation of the XylG protein. However, when adaptive evolution was performed in this study, a *xylG* revertant was not identified. Instead, the adaptive mutations were identified in the *xylR* gene (Table 2).

An increase in the transcription of xylose metabolizing genes leads to the expression of metabolic enzymes, which can lead to an increase in xylulose-5-phosphate in the pentose phosphate pathway, which further increases glyceraldehyde-3-phosphate metabolic pool. Moreover, substantially more NADPH can be generated in the pentose phosphate pathway compared to the NADH produced in glycolysis. Differences in the reoxidation process between NADPH and NADH may change the pattern of metabolite fermentation (e.g., a decrease in succinate and/or an increase in ethanol) in the evolved strains (Table 1). Additionally, we cannot rule out the possibility that changes in the intracellular concentration of metabolites and/or coenzymes such as NADPH may induce allosteric regulation of one or more enzymes in the lower part of glycolysis or acetyl-CoA metabolism, which might affect the pattern of metabolite fermentation.

We then tested whether the *carB* gene mutation, which was additionally identified in the adaptively evolved JH019 strain, affected the xylose consumption rate. Notably, the JH001 strain exhibited growth delays during D-xylose consumption (Fig. 3D). However, when the *carB* gene was deleted in the JH001 strain, faster cell growth with higher OD values were observed in the D-xylose medium (Fig. S4). It is still unclear how the *carB* gene mutation is related to the anaerobic growth and fermentation profiles of JH019 cells, a strain evolved from BL21(DE3). Strain BL21(DE3) is known to have defects in anaerobic metabolism due to its mutation in the *fnr* gene. Specifically, the DE3 episome is inserted within the genes encoding the molybdenum transport system in this strain [36]. Moreover, carbamoyl phosphate, which is generated by carbamoyl phosphate synthetase (encoded by *carB*), is known to be required for hydrogenase maturation [37] and may be linked to redox metabolism alterations.

In summary, to improve the anaerobic D-xylose consumption rate of the BL21(DE3) strain, which has a slower xylose consumption rate compared to other wild-type *E. coli* strains, *xylR* adaptive mutations were obtained through experimental evolution. Our study demonstrated that *E. coli* strains can rapidly acquire genome mutations that enable them to consume D-xylose sugars even with a single base substitution, suggesting that a variety of variants may exist at the species or strain level. The gain-of-function point mutation in the *xylR* gene was introduced into a new background through target-mismatched CRISPR/Cas9 genome editing technology, resulting in *E. coli* strains capable of efficient D-xylose consumption under anaerobic conditions. Therefore, our findings provide an important basis for the development of fermentation biotechnology.

## Methods

### Strains and culture conditions

The strains and plasmids used in this study are listed in Supplementary Table 1. Bacterial seed cultures were grown in 5 ml of LB broth (Cat. No. LB-05, LPS solution, Daejeon, Korea) at 37 °C with constant mixing at 180 rpm. One milliliter of seed culture was used to inoculate a 125 ml serum vial with a butyl rubber stopper containing 100 ml of fermentation medium, as described previously [38]. The medium contained the following components (per liter): yeast extract = 5 g; NaHCO_3_ = 10 g; NaH_2_PO_4_·H_2_O = 8.5 g; K_2_HPO_4_ = 15.5 g. Yeast extract (Cat. No. 212750) was purchased from Becton Dickinson (Sparks, MD, United States). NaHCO_3_ (Cat. No. S6014), NaH_2_PO_4_·H_2_O (Cat. No. S9638), and K_2_HPO_4_ (Cat. No. P3786) were purchased from Sigma-Aldrich (St. Louis, MO, United States). The headspace of the fermentation bottles was filled with nitrogen gas, and sodium sulfide (final concentration 1 mM) was added to quench the dissolved oxygen, thus yielding strictly anaerobic conditions. Bacterial cells were grown anaerobically at 37 °C with constant mixing at 180 rpm. Additionally, 25 mM D-glucose (Cat. No. 64220S0650, JUNSEI, Tokyo, Japan), 25 mM D-xylose (Cat. No. 25190S0401, JUNSEI, Tokyo, Japan), or 12.5 mM D-glucose + 12.5 mM D-xylose were incorporated as carbon sources.

For serial transfer, 1 mL of seed cultures were added to a 100 mL fermentation medium containing 12.5 mM D-glucose and 12.5 mM D-xylose. Cells were grown anaerobically at 37 °C with constant mixing at 180 rpm. Cell growth and residual D-glucose and D-xylose were monitored throughout the experiment. Once D-glucose and D-xylose were fully depleted, serial passages were performed by diluting the culture to a 1:100 ratio in 100 mL of a fermentation medium containing 12.5 mM D-glucose and 12.5 mM D-xylose. To obtain adapted strains, the cultures were spread on LB plates to obtain pure isolates from fermentation broth.

### Analytical procedures

Sugar and metabolite concentrations were measured using high-performance liquid chromatography (Waters 410 RI Monitor, Waters; MA, United States) using an Aminex HPX-87H column (300 mm × 7.8 mm, BioRad, Hercules, CA, United States) as described previously [39]. The cell culture broth was then centrifuged, after which the supernatant was passed through a 0.2 μm syringe filter. The column was isocratically eluted at 47 °C with a flow rate of 0.5 mL min^− 1^ using 0.01 N H_2_SO_4_ (Cat. No. 258105-500 ml, Sigma-Aldrich, St. Louis, MO, United States). Cell growth was monitored by measuring the optical density of the culture media at 600 nm using an Ultraspec 8000 spectrophotometer (GE Healthcare, Uppsala, Sweden). The cell cultures were diluted using phosphate buffered saline to measure the optical density. The maximum sugar consumption rate was calculated as the amount of sugar consumed divided by the fermentation time (mM/h) in the section where sugar was consumed most rapidly.

### Genome sequencing

The genomic DNA of different *Escherichia coli* strains was purified with the Wizard Genomic DNA Purification Kit (Cat. No. A1120, Promega, Madison, WI, United States). The genome sequences of the adaptively evolved strains were obtained with an Illumina HiSeq 2500 sequencer. Pretreatment of the reads, reference mapping, and variant detection were carried out using the Genome Analysis Tool Kit (GATK). Reads shorter than 50 nt were filtered out after quality trimming using Trimmomatic Version 0.36 (Table 2). The genome sequences of *E. coli* BL21(DE3) (CP001509.3) were used for reference mapping. Genome sequencing data were deposited in the NCBI BioProject database under accession number PRJNA689415. Sanger sequencing was conducted to confirm the *xylR* sequence. *xylR* was amplified using the xylR_250F and xylR_100R primer pairs in Supplementary Table 2.

### Genome editing

Mutations were transferred to other strains via standard P1 transduction [40]. To obtain the Δ*xylR* mutant strain, P1 *vir* phage lysates of kanamycin-resistant strain BW25113 Δ*xylR* (JW3541) from the KEIO collection were used to transduce the BL21(DE3) strain to generate JH003 strain.

To introduce *xylR* C91A or C361T point mutation, oligo-directed mutagenesis was performed, and negative selection was carried out using the CRISPR-Cas9 system, as described in a previous study [30]. The genomic point mutations were confirmed via Sanger sequencing. Next, the CRISPR-Cas9 gene in the genome of the edited *E. coli* cells was removed through P1 transduction, and temperature-sensitive sgRNA plasmids were removed by incubating the cells at 42 °C.

To introduce a Δ*carB* mutation, P1 *vir* phage lysates of kanamycin-resistant strain BW25113 Δ*carB* (JW0031) from the KEIO collection were used to transduce strains BL21(DE3) and JH001 as recipient cells to generate JH042 and JH044, respectively.

### Transcript analyses

The transcription of the *xylA* and *xylF* genes was confirmed using quantitative real-time PCR (qRT-PCR). The BL21(DE3) wild type strain and adapted strains were grown for 8 h under anaerobic conditions in fermentation media containing 12.5 mM D-glucose and 12.5 mM D-xylose or 25 mM D-xylose at 37 °C, and RNA was isolated using the RNeasy® Mini Kit (Cat. No. 74104, Qiagen, Hilden, Germany). qRT-PCR primer sequences for target genes were designed using the IDT PrimerQuest®Tool (Supplementary Table 2). qRT-PCR was conducted using a CFX Connect system (BioRad, Hercules, California, United States) using the RealHelix™ qRT-PCR Kit (Cat. No. QRT-S500, Nanohelix, Daejeon, Korea). Five Nanograms of total RNA was used in qRT-PCR reactions under the following conditions: cDNA synthesis (50 °C, 40 min); denaturation (95 °C, 12 min); amplification for 40 cycles (95 °C, 20 s; 60 °C, 1 min). The raw fluorescence data were normalized against the expression level of the 16S ribosomal RNA and their corresponding expression levels in the BL21(DE3) wild-type strain.

## Supplementary Information

**Additional file 1 **: **Figure S1.** Cell growth and D-xylose consumption profiles of wild-type *E. coli* strains in anaerobic conditions. Only D-xylose (25 mM) was added as a carbon source in the fermentation medium. (A) BL21(DE3); (B) BW25113; (C) C strain; (D) MG1655. **Figure S2.** Molecular structure of XylR dimer with D-xylose (PBD ID: 4FE7). Green, DNA binding domain; Pink, D-xylose; Red, Q31 (Q31K mutation site identified in JH001 strain); Blue, A247 (A247V mutation site identified in JH019 strain). **Figure S3.** Anaerobic cell growth and sugar consumption profiles of JH003 (BL21(DE3) Δ*xylR*) strain. (A) Cells grown in a fermentation medium containing both D-glucose (12.5 mM) and D-xylose (12.5 mM), (B) cells grown in a fermentation medium containing D-xylose only (25 mM). **Figure S4.** Anaerobic cell growth sugar consumption profiles of Δ*carB* strains. (A) JH042 (= BL21(DE3) Δ*carB*) grown in a fermentation medium containing both D-glucose (12.5 mM) and D-xylose (12.5 mM), (B) JH042 in D-xylose only (25 mM) medium, (C) JH044 (= JH001 Δ*carB*) in D-glucose (12.5 mM) + D-xylose (12.5 mM) medium, (D) JH044 in D-xylose only (25 mM) medium. **Figure S5**. Anaerobic cell growth and sugar consumption profiles of JH036 (BL21(DE3) *xylR*^C361T^) strain in a fermentation medium containing D-glucose (12.5 mM) and D-xylose (12.5 mM). **Table S1.** *E. coli* strains and plasmids used in this study. **Table S2.** Primers used in this study.

## Data Availability

Genome sequencing data were deposited in the NCBI BioProject database under accession number PRJNA689415 (https://www.ncbi.nlm.nih.gov/search/all/?term=PRJNA689415).
