# Supplementary material for: Efficient anaerobic consumption of D-xylose by E. coli BL21(DE3) via xylR adaptive mutation
Source: BMC Microbiol. 2021 Dec 6;21:332. doi: 10.1186/s12866-021-02395-9 (PMC8647362; doi:10.1186/s12866-021-02395-9)
Supplement: Supplementary file 1 — Additional file 1 : Figure S1. Cell growth and D-xylose consumption profiles of wild-type E. coli strains in anaerobic conditions. Only D-xylose (25 mM) was added as a carbon source in the fermentation medium. (A) BL21(DE3); (B) BW25113; (C) C strain; (D) MG1655. Figure S2. Molecular structure of XylR dimer with D-xylose (PBD ID: 4FE7). Green, DNA binding domain; Pink, D-xylose; Red, Q31 (Q31K mutation site identified in JH001 strain); Blue, A247 (A247V mutation site identified in JH019 strain). Figure S3. Anaerobic cell growth and sugar consumption profiles of JH003 (BL21(DE3) ΔxylR) strain. (A) Cells grown in a fermentation medium containing both D-glucose (12.5 mM) and D-xylose (12.5 mM), (B) cells grown in a fermentation medium containing D-xylose only (25 mM). Figure S4. Anaerobic cell growth sugar consumption profiles of ΔcarB strains. (A) JH042 (= BL21(DE3) ΔcarB) grown in a fermentation medium containing both D-glucose (12.5 mM) and D-xylose (12.5 mM), (B) JH042 in D-xylose only (25 mM) medium, (C) JH044 (= JH001 ΔcarB) in D-glucose (12.5 mM) + D-xylose (12.5 mM) medium, (D) JH044 in D-xylose only (25 mM) medium. Figure S5. Anaerobic cell growth and sugar consumption profiles of JH036 (BL21(DE3) xylRC361T) strain in a fermentation medium containing D-glucose (12.5 mM) and D-xylose (12.5 mM). Table S1. E. coli strains and plasmids used in this study. Table S2. Primers used in this study. [file 12866_2021_2395_MOESM1_ESM.pdf]

Supplementary Table 1. *E. coli* strains and plasmids used in this study.

Name	Relevant genotypes or characteristics	Reference or source
E. coli K-12		
BW25113	Δ(*araD-araB*)*567* Δ*lacZ4787*(::*rrnB-3*) λ^- *rph-1* Δ(*rhaD-rhaB*)*568 hsdR514*	Laboratory stock
MG1655	K-12 F^- λ^- *ilvG*$^-$ *rfb-50 rph-1*	Laboratory stock
W3110	F^- λ^- rph-1 INV(*rrnD*, *rrnE*)	Laboratory stock
JW0031	BW25113, Δ*carB*::FRT-KmR-FRT	Keio collection
JW3541	BW25113, Δ*xylR*::FRT-KmR-FRT	Keio collection
JH053	MG1655, *araBAD*::P_{BAD}-*cas9*-*bet*-CmR	This study
JH057	MG1655, *araBAD*::P_{BAD}-*cas9*-*bet*-CmR, *xylR* C91A (Q31K)	This study
JH061	MG1655, *xylR* C91A (Q31K)	This study
E. coli B		
BL21(DE3)	*E. coli* str. B F^- *ompT gal dcm lon* $hsdS_B(r_B^- m_B^-)$ λ (DE3 [*lacI lacUV5-T7p07 ind1 sam7 nin5*]) [$malB^+$]$_{K-12}$(λ^S)	Laboratory stock
JH001*	BL21(DE3), *xylR* C91A (Q31K)	This study
JH003	BL21(DE3), *xylR*::KmR	This study
JH010	BL21(DE3), *araBAD*::P_{BAD}-*cas9*-*bet*-CmR	This study
JH019*	BL21(DE3), *xylR* C740T (A247V), *carB*::IS1	This study
JH031	BL21(DE3), *araBAD*::P_{BAD}-*cas9*-*bet*-CmR, *xylR* C91A (Q31K)	This study
JH035	BL21(DE3), *xylR* C91A (Q31K)	This study
JH042	BL21(DE3), *carB*::KmR	This study
JH044	BL21(DE3), *xylR* C91A (Q31K), *carB*::KmR	This study
JH046	BL21(DE3), *xylR* C740T (A247V), *carB*::KmR	This study
E. coli C		
C strain		ATCC8739
Phage		
P1 *vir*	*vir* mutations	Laboratory stock
Plasmid		
pCP20	Temperature-sensitive plasmid with an FLP recombinase capable of recognizing the FRT sequence, ApR	Laboratory stock
pKD46	pSC101 ori^{ts}, *araC*, λ *red* genes, AmpR	Laboratory stock
pHK463	pSC101 ori^{ts}, *araC*, λ *bet* gene, AmpR	Laboratory stock
pJH003	pSC101 ori^{ts}, sgRNA target (^{84}ATATTTACCCGCGTCACAAT^{103} in *xylR*), SpR, AmpR	This study
pJH008	pSC101 ori^{ts}, sgRNA target (^{355}GTTAACCGCTTTGCTTTTTA^{374} in *xylR*), SpR, AmpR	This study

* Whole genomic sequencing was performed.

Supplementary Table 2. Primers used in this study.

**Name**	**Sequence (5′ → 3′)**	**Description**
xylR_250F	GCAGCATGCGTGATTGGCGGCACCAGCC	Amplification of *xylR* gene
xylR_100R	GAGCTACGAGCCTGAAGCGTTGTTGGTG	
carB_250F	GATCACCGCCCAGAACCACGGTTTTGCG	Amplification of *ysaB* gene
carB_100R	GATTCCCCAAAAGATTTACTTAATGGGC	
Cas9_400up	AAGTTGAAGGGTAGTCCAGAAGATAACG	Confirmation of *araBAD* cassette
AraD_500dn	CCAGCCAGAAGGAGACTTCTGTCCCTTG	
xylA_RT_F	CTCGATCGCGTTCGTTATG	RT-qPCR for *xylA* gene
xylA_RT_R	GCTCTTCCATACGCTTACC	
xylF_RT_F	ATTCTACTCACCCTTTGCAC	RT-qPCR for *xylF* gene
xylF_RT_R	GTTCAAGACGGAGATCATCAA	
16S-RLT	CAGCAGCCGCGGTAATAC	RT-qPCR for 16S rRNA gene
16S-RTL	ACCAGGGTATCTAATCCTGT	
Sm_ATG_Out	GATACTGGGCCGGCAGGCGCTCCATTGCCC	sgRNA vector construction
Sm_TAA_Out	GCAATGGAGCGCCTGCCGGCCCAGTATCAG	
xylR92cc_F	ATATTTACCCGCGTCACAATGTTTTAGAGCTAGAAATAGCAAG	pJH003 construction
xylR92cc_R	ATTGTGACGCGGGTAAATATACTAGTATTATACCTAGGACTG	
xylRc91a	AAGGCGTAGGGGAATATTTAAAGGCGTCACAATCGGAATGG	*xylR* ^{91}C to A mutagenic oligo
xylR363g_F	GTTAACCGGTTTGCTTTTTAGTTTTAGAGCTAGAAATAGCAAG	pJH008 construction
xylR363g_R	TAAAAAGCAAACCGGTTAACACTAGTATTATACCTAGGACTG	
xyolRc361t	TAAAAGAGAAAGGCGTTAACTGCTTTGCTTTTTATGGTCTT	*xylR* ^{361}*C to T mutagenic oligo*

Supplementary Figure 1

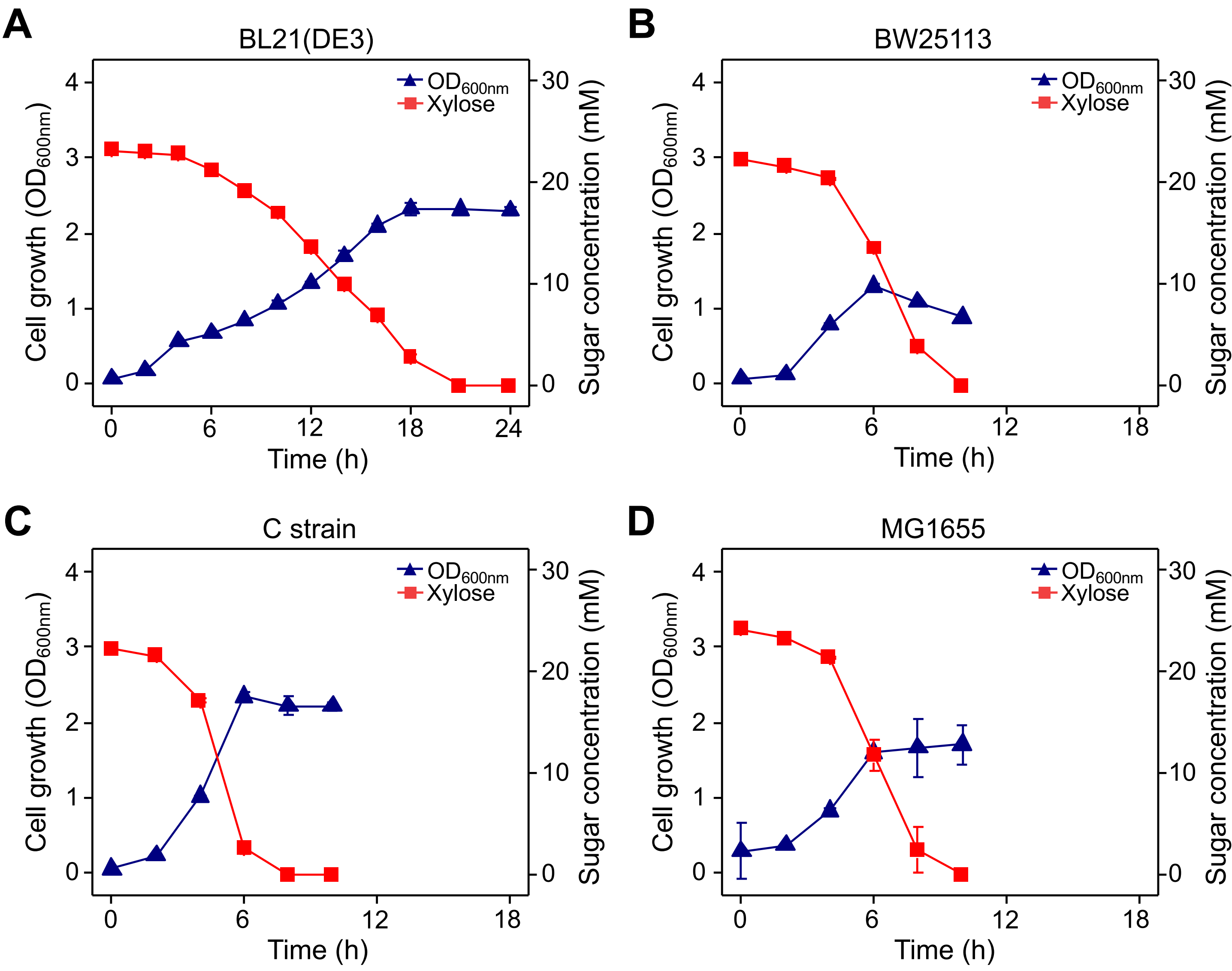

Figure S1. Cell growth and D-xylose consumption profiles of wild-type *E. coli* strains in anaerobic conditions. Only D-xylose (25 mM) was added as a carbon source in the fermentation medium. (A) BL21(DE3); (B) BW25113; (C) C strain; (D) MG1655.

Supplementary Figure 2

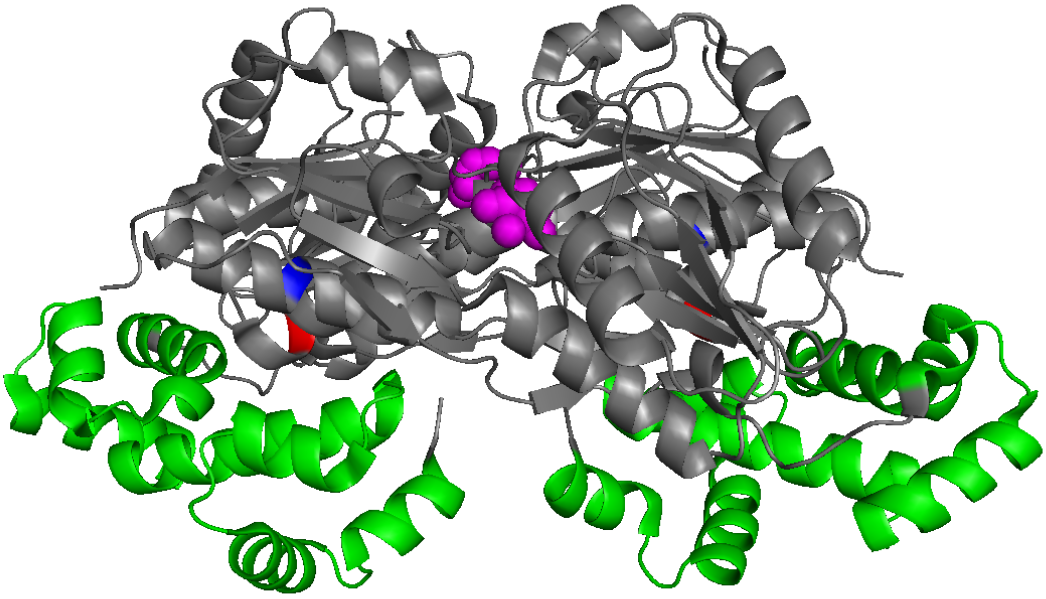

Figure S2. Molecular structure of XylR dimer with D-xylose (PBD ID: 4FE7). Green, DNA binding domain; Pink, D-xylose; Red, Q31 (Q31K mutation site identified in JH001 strain); Blue, A247 (A247V mutation site identified in JH019 strain).

Supplementary Figure 3

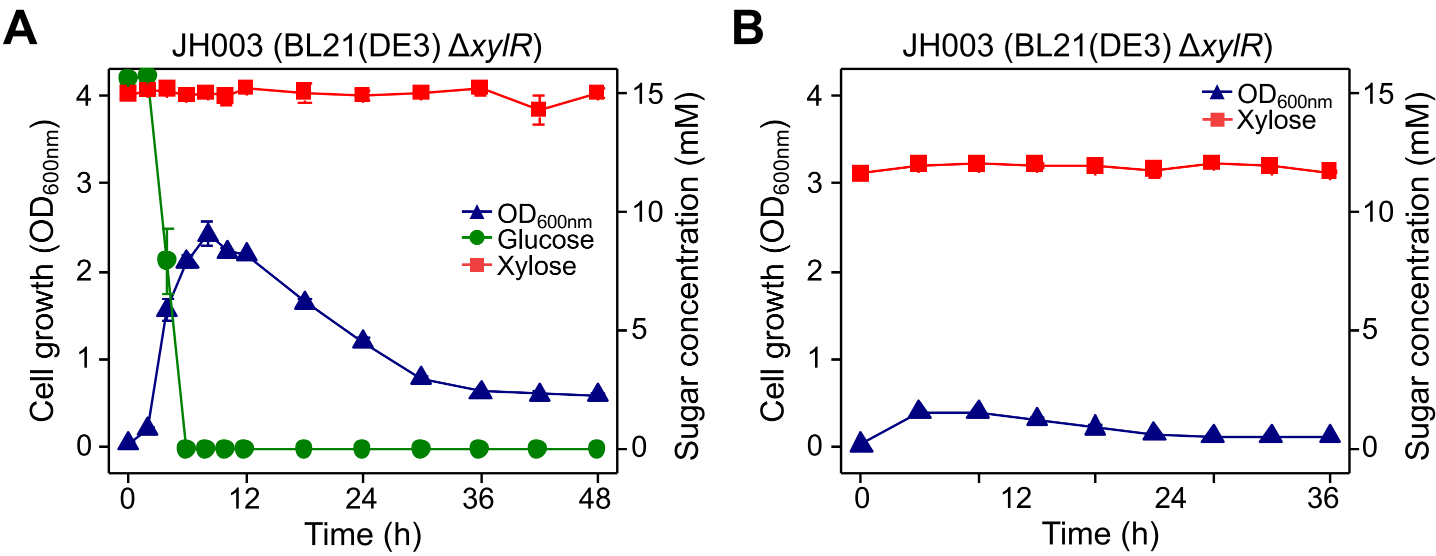

Figure S3. Anaerobic cell growth and sugar consumption profiles of JH003 (BL21(DE3) Δ*xylR*) strain. (A) Cells grown in a fermentation medium containing both D-glucose (12.5 mM) and D-xylose (12.5 mM), (B) cells grown in a fermentation medium containing D-xylose only (25 mM).

Supplementary Figure 4

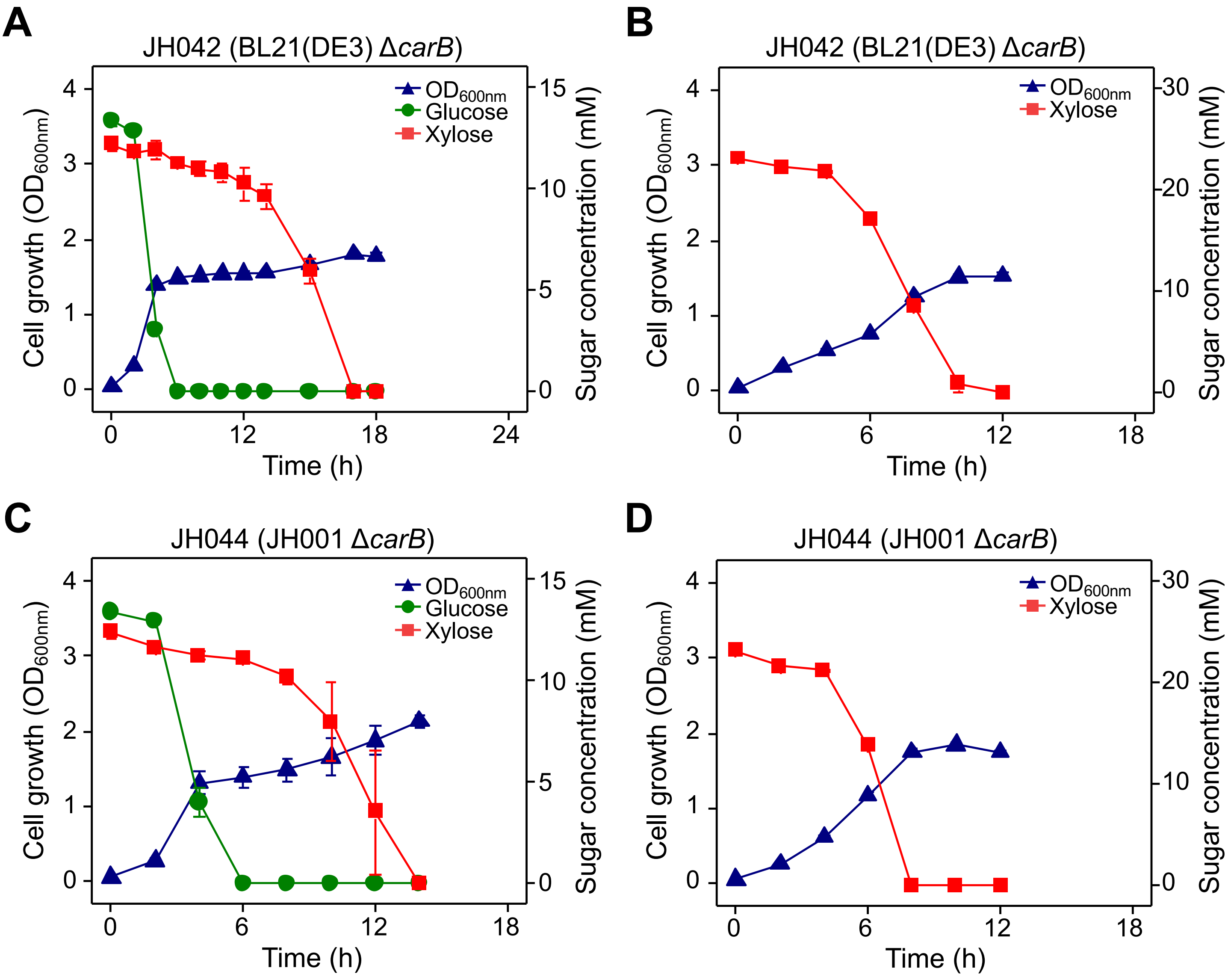

Figure S4. Anaerobic cell growth sugar consumption profiles of Δ*carB* strains. (A) JH042 (= BL21(DE3) Δ*carB*) grown in a fermentation medium containing both D-glucose (12.5 mM) and D-xylose (12.5 mM), (B) JH042 in D-xylose only (25mM) medium, (C) JH044 (= JH001 Δ*carB*) in D-glucose (12.5 mM) + D-xylose (12.5 mM) medium, (D) JH044 in D-xylose only (25mM) medium.

Supplementary Figure 5

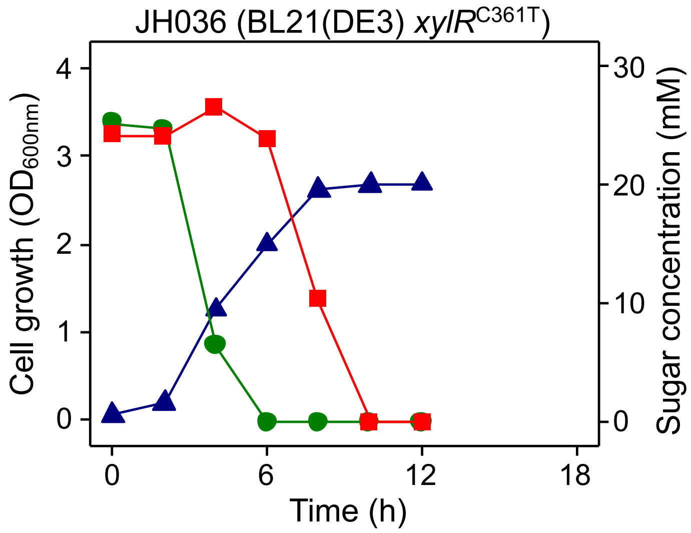

Figure S5. Anaerobic cell growth and sugar consumption profiles of JH036 (BL21(DE3) *xylR*^{C361T}) strain in a fermentation medium containing D-glucose (12.5 mM) and D-xylose (12.5 mM).
